# Blood gene expression network expression strongly relates to brain amyloid burden

**DOI:** 10.1002/alz.70982

**Published:** 2025-12-19

**Authors:** Vaibhav A. Janve, Mabel Seto, Reisa A. Sperling, Paul S. Aisen, Robert A. Rissman, Mary Ellen I. Koran, Logan Dumitrescu, Rachel F. Buckley, Timothy J. Hohman, A4 and LEARN Study Group

**Affiliations:** ^1^ Vanderbilt Memory & Alzheimer's Center Vanderbilt University Medical Center Nashville Tennessee USA; ^2^ Vanderbilt Genetics Institute Vanderbilt University Medical Center Nashville Tennessee USA; ^3^ Center for Alzheimer Research and Treatment Brigham and Women's Hospital Harvard Medical School Boston Massachusetts USA; ^4^ Center for Alzheimer's Research and Treatment Brigham and Women's Hospital, Massachusetts General Hospital Harvard Medical School Boston Massachusetts USA; ^5^ Alzheimer's Therapeutic Research Institute Keck School of Medicine University of Southern California San Diego California USA; ^6^ Department of Radiology Vanderbilt University Medical Center Nashville Tennessee USA; ^7^ Department of Neurology Massachusetts General Hospital Harvard Medical School Boston Massachusetts USA; ^8^ Melbourne School of Psychological Sciences University of Melbourne Melbourne Victoria Australia; ^9^ Alzheimer's Clinical Trial Consortium Boston Massachusetts USA

**Keywords:** amyloid PET, bulk RNA‐seq, CD4+ activated memory T cells, CIBERSORTx, deconvolution, digital cytometry, gene expression, histone, natural killer cells, preclinical AD, p‐tau217, WGCNA, whole blood

## Abstract

**INTRODUCTION:**

Amyloid deposition occurs decades before symptoms emerge in Alzheimer's disease (AD). We leveraged blood transcriptomics and positron emission tomography (PET) measures of amyloidosis to identify gene networks in the blood that relate to amyloid burden in the brain.

**METHODS:**

Whole‐blood RNA sequencing and amyloid PET were leveraged from 1739 cognitively unimpaired participants in the Anti‐Amyloid Treatment in Asymptomatic Alzheimer's Disease (A4) study. Linear regression related gene module expression to amyloid covarying for age, sex, education, and *apolipoprotein E (APOE) ε2 and ε4 genotypes*.

**RESULTS:**

Of the 18 gene modules, one histone gene cluster module was associated with amyloid (*β* = −0.55, false discovery rate‐adjusted *p* value = 0.029). We also observed nominal associations for the predicted proportion of activated natural killer (NK) cells (*β* = −0.454, *p* = 0.02) and CD4+ activated memory T cells (*β* = −0.169, *p* = 0.03) with amyloid deposition.

**DISCUSSION:**

Our results implicate the histone gene cluster on chromosome 6 and immune cell proportions as blood correlates of brain amyloid deposition in preclinical AD.

**Highlights:**

- Higher expression of network module with histone gene cluster on chromosome 6 associated with lower amyloid levels.
- Four histone genes, *H1‐5*, *H3C3*, *H2BC3*, *H2AC14*, and *RRM2*, emerged as key genes driving this association, where *H1‐5* emerged as a hub gene for this module.
- Pathways, including nucleosome assembly and DNA damage, were enriched in the histone module.
- A higher fraction of activated NK and activated CD4+ T cells was related to lower amyloid burden.

## 1 BACKGROUND

AD is characterized by two primary neuropathologies: amyloid beta (Aβ) plaques outside of the neuron and neurofibrillary tangles within the neuron. The pathogenesis of the disease includes a long prodrome in which the neuropathology is beginning to accumulate in the absence of clinical impairment. Aβ accumulation occurs 17 to 23 years before the clinical symptoms of AD appear and serves as a sensitive biomarker that strongly predicts future AD progression.^1^, ^2^, ^3^ Blood‐based biomarkers of amyloid deposition are increasingly used to monitor early brain changes.^4^ The goal of this project was to nominate novel peripheral blood biomarkers and highlight novel molecular pathways that change in the earliest stages of the disease.

Numerous studies have highlighted notable shifts in the transcriptomic profile in the AD brain,^5^, ^6^ while fewer have characterized alterations in the blood of AD patients.^7^, ^8^, ^9^ Most blood transcriptomic studies of AD have been small and focused entirely on differences between AD cases and controls. The recently completed Anti‐Amyloid Therapy in Asymptomatic Alzheimer's Disease clinical trial (A4) study has provided an unprecedented opportunity to explore blood transcriptomic signatures of an important AD endophenotype–brain amyloid burden by collecting amyloid imaging and blood transcriptomic data in a large sample (*N* = 1739) of cognitively unimpaired older adults.

Our work leverages the blood transcriptome to better understand the earliest biological changes in AD. Specifically, we have built whole‐blood transcript co‐expression networks to explore novel molecular drivers of early amyloid changes. Additionally, we utilize gene set enrichment analysis and cell‐type deconvolution algorithms to identify biologically relevant pathways and cell populations in the blood that most strongly relate to brain amyloid changes in the brain. We hypothesize that gene expression changes in the blood are detectable early in disease and reflect fundamental shifts in biological pathways that are relevant to disease progression.

## 2 METHODS

### 2.1 Study participants

This study included 1739 participants (Table 1) from the screening dataset of the A4 study and the companion observational Longitudinal Evaluation of Amyloid Risk and Neurodegeneration (LEARN) study with available brain amyloid positron emission tomography (PET) imaging (^18^F‐Florbetapir) and whole‐blood RNA sequencing (RNA‐seq) data. Participants aged 65 to 85 with normal cognitive function were recruited from over 60 sites across the United States, Canada, Japan, and Australia. The A4 study screened 6763 individuals, enrolling and randomizing 1169 participants with elevated Aβ levels. The LEARN study enrolled 538 participants who did not meet the amyloid criteria for A4.^10^

**TABLE 1:** Participant demographics.

	A4/LEARN study participants
Number of participants	1739
Female sex	62%
Age (years)	71.3 ± 4.7
Education (years)	16.5 ± 2.7
*APOE* ε4 positive	36%
*APOE* ε2 positive	13%
Race	
White	1665
African American	50
Asian	27
Multiple or unknown	29
Ethnicity	
Hispanic	51
Non‐Hispanic	1688
Amyloid PET positive	32%

Abbreviation: A4, Anti‐Amyloid Treatment in Asymptomatic Alzheimer's Disease; LEARN, Longitudinal Evaluation of Amyloid Risk and Neurodegeneration; PET, positron emission tomography.

Informed consent was obtained from all participants as part of the parent study, and the Vanderbilt University Institutional Review Board approved the secondary analysis of these data for the present manuscript.

### 2.2 Amyloid PET imaging

Amyloid PET scans were conducted using standardized protocols across all participating sites to ensure consistency and reliability of the data.^11^ Participants received an intravenous injection of a radiotracer, florbetapir (^18^F‐AV‐45), which binds to amyloid plaques. After an uptake period of 50 to 70 min, participants underwent a 20‐ to 30‐min PET scan. The resulting images were used to quantify amyloid burden in various brain regions as reported previously.^12^, ^13^ A global cortical standardized uptake value ratio (SUVR) was quantified using the whole cerebellum as the reference region. Additional details on acquisition and processing pipelines can be found at a4studydata.org.

**RESEARCH IN CONTEXT**

1. **Systematic review**: The authors reviewed the literature using traditional (e.g., PubMed) sources and meeting abstracts and presentations. While large blood transcriptomic studies in preclinical AD studying brain amyloid are limited, numerous publications highlight transcriptomic profile shifts in AD brain and blood‐based biomarkers of amyloid deposition. These relevant works are appropriately cited.
2. **Interpretation**: Our findings provide support to early involvement of epigenetic regulation in AD pathogenesis and highlight genes, cell types, and molecular pathways that change in the earliest stages of AD.
3. **Future directions**: The manuscript lays the groundwork for further investigations of blood–brain compartment interactions and to improve preclinical prediction of future cognitive decline in older adults using blood transcriptomics, gene network alterations, and cell‐type‐specific peripheral changes involving immune and epigenetic regulation.

### 2.3 Plasma phosphorylated tau 217 (p‐tau217) assay

Plasma samples were processed at the Eli Lilly and Company (Lilly) Diagnostics Laboratory. The p‐tau217 levels were measured using an electrochemiluminescent immunoassay, with sample preparation automated by the Tecan Fluent workstation and detection performed on the MSD Sector S Imager 600 MM.^14^

### 2.4 RNA sequencing protocol

2.5 mL of whole‐blood samples were collected in Paxgene Tubes, frozen on site, shipped on dry ice, and stored at −80°C. Total RNA was extracted from whole blood using the QIASymphony RNA Kit (QIAGEN, 931636), and both ribosomal RNA and hemoglobin were depleted with the NEBNext Globin and rRNA Depletion Kit (New England BioLabs, Inc., E7750). Library preparation was completed using the NEBNext Ultra Directional Library Prep Kit (New England BioLabs, Inc., E7420) before sequencing was performed using 151 base pair (bp) paired‐end reads on an Illumina NovaSeq 6000 (Illumina), targeting an average of 60 million reads per sample, and 1798 samples were sequenced in 19 batches. Quality control (QC) reports for the sequencing quality and the per‐sample yield were provided along with a demultiplexed FASTQ containing the passing filter reads. The mean (±SD) RNA integrity number (RIN) score for the 1798 samples was 7.61 (± 1.10), while the RIN score for the control sample was 10.0. Sixteen batches were analyzed on the LabChip GX Touch nucleic acid analyzer and provided both well and peak tables. Three batches (7, 14, and 19) were analyzed on Agilent Tapestation and provided only well tables with RIN scores.

### 2.5 RNA sequencing quality control

Raw counts were loaded into R (version 4.3.1). The initial dataset consisted of 1798 samples and 62,754 genes. Missing RIN scores (*n* = 57) were estimated using multivariate imputation by chained equations with predictive mean matching method implemented in MICE R package. Samples with missing apolipoprotein E (*APOE)* genotypes (*n* = 26) were determined from genome‐wide association study data. Ten samples with RIN < 4, 25 samples with any missing covariates, and 11 sample outliers on sex genes (*XIST* and *UTY*) were excluded, resulting in 1752 samples. Next, 92 genes without GC content or gene length were removed. For subsequent steps, the autosomal, X, and Y gene counts were processed separately. Any genes for which there were <1 Counts Per Million (CPM) in 50% of samples were removed, resulting in 1752 samples and 20,720 genes (19,611 autosomal, 1050 X, and 59 Y chromosome genes). The autosomal gene expression principal components (PCs) were assessed for outliers (greater or less than 5 SD from mean) on first two PCs; 13 samples were removed. Conditional quantile normalization was performed using the cqn package (version 1.30.0) using GC content, the percentage of Guanine (G) and Cytosine (C) bases, obtained from Ensembl via the biomaRt package (version 2.56.1) and gene lengths obtained from featureCounts. Next, gene counts were Winsorized on a per‐gene basis (>5 SD), and outliers were removed. *Limma‐voom* was used to iteratively adjust for the effects of the following covariates: batch, age, sex, percent of aligned reads, percent of coding bases, race, and any genes with 0 variance (*n* = 2) were dropped during this iterative process. The final dataset consisted of 1739 samples and 20,718 genes.

### 2.6 Gene co‐expression network analysis: WGCNA

Processed gene expression counts were used to construct gene co‐expression network with Weighted Gene Co‐expression Network Analysis (WGCNA) (version 1.72‐5) using default parameters, and co‐expression modules were determined as previously described.^15^, ^16^, ^17^ In brief, a similarity matrix was calculated based on Pearson's correlation for each pair of genes across all samples. Next, the similarity matrix was transformed into an adjacency matrix. Then the topological overlap matrix and the corresponding dissimilarity matrix were computed. Finally, a dynamic tree cut algorithm determined the gene co‐expression modules. The following parameters were used: a soft threshold power of 15 for scale‐free network construction, networkType = “signed,” deepSplit = 2, minModuleSize = 30, maxBlockSize = 4000, and mergeCutHeight = 0.25. Hub genes, genes with the highest degree of connectivity within the module, were identified for each module using chooseTopHubInEachModule() function in WGCNA. Gene network modules were visualized with Cytoscape software (version 3.10.2).

### 2.7 Cell fraction estimation

Abundances (cell fractions) for 22 cell types were determined from gene expression data using the deconvolution‐based CIBERSORTx^18^ analytical tool using the LM22 leukocyte gene signature matrix to estimate the cell‐type composition from whole‐blood data.^19^ Based on the transcriptomic signature, this algorithm can estimate the abundance of 22 blood cell types: naïve B cells, memory B cells, plasma cells, CD8 T cells, naïve CD4 T cells, memory resting CD4 T cells, memory activated CD4 T cells, follicular helper T cells, regulatory T cells, gamma delta T cells, resting natural killer (NK) cells, activated NK cells, monocytes, macrophages M0, macrophages M1, macrophages M2, resting dendritic cells, activated dendritic cells, resting mast cells, activated mast cells, eosinophils, and neutrophils.

### 2.8 Statistical analysis – associations with amyloid PET

We performed two sets of linear regression analyses covarying for age, sex, education, and *APOE* ε2 and ε4 genotype status. The first analysis regressed amyloid burden onto gene modules to identify gene modules that relate to amyloid deposition. The second set of analyses regressed amyloid burden on cell fraction estimates to identify cell‐type associations. Correction for multiple comparisons was performed using the Benjamani–Hochberg false discovery rate (FDR) procedure.

### 2.9 Gene set enrichment analysis

We further characterized each gene co‐expression module by performing gene set enrichment analysis, based on gene sets available on the Gene Ontology (GO) and Kyoto Encyclopedia of Genes and Genomes (KEGG) databases (https://geneontology.org/).^20^, ^21^, ^22^ Gene set enrichment analysis (specifically, over‐representation analysis) was performed in clusterProfiler, where each gene module was analyzed with all 20,718 measured genes set as background for probability estimates. Hypergeometric tests were used to estimate the likelihood that the module genes are overrepresented in the list of genes annotated in the given GO/KEGG category. Correction for multiple comparisons was again completed leveraging the FDR procedure across all pathways evaluated.

### 2.10 Sensitivity analyses

To compare our results with known blood biomarkers of brain amyloidosis, we performed sensitivity analyses evaluating whether significant gene module or cell‐type associations with brain amyloid burden remained significant when covarying for blood p‐tau217 (*n* = 724).

## 3 RESULTS

Participant characteristics are presented in Table 1. All participants were cognitively unimpaired at baseline, 71 years of age on average, and highly educated, and nearly two‐thirds were female. A third of participants were amyloid positive.

### 3.1 Gene co‐expression network module associations with brain amyloid

**FIGURE 1:**
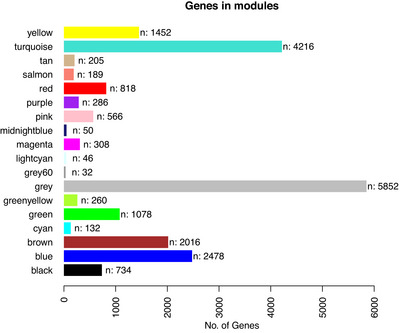
Gene expression modules determined with Weighted Gene Co‐expression Network Analysis analysis. The gene module is presented along the *y*‐axis. The number of genes is presented along the *x*‐axis.

Gene co‐expression network analysis resulted in the identification of 18 gene modules that were then used in association analyses (Figure 1). Comprehensive association results are presented in Table S1. One module, grey60, showed an association with brain amyloid burden that survived correction for multiple comparisons (*p* = 1.60E‐03, *p_FDR_* = 2.87E‐02) presented in Table 2.

**TABLE 2 alz70982-tbl-0002:** WGCNA‐derived gene expression network modules associated with brain amyloid at false discovery rate (FDR) significance levels *p_FDR_* < 0.05.

Module	No. of genes	*β*	*P*	*p_FDR_*
MEgrey60	32	−0.547	1.60E‐03	2.87E‐02

**FIGURE 2:**
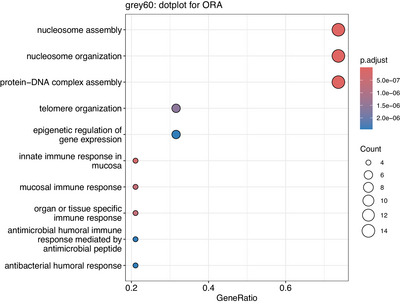
Top Gene Ontology (GO) pathways in the grey60 module. The GO term is presented along the *y*‐axis. The gene ratio is presented along the *x*‐axis. Dot size represents the count, and the color of the dot represents the *p* value after adjustment for multiple comparisons.

The grey60 module included 32 genes in which higher expression of the module was related to lower levels of amyloid deposition in the brain (*β* = −0.55). All individual transcript associations from the grey60 module are presented in Table S2, and transcriptome‐wide amyloid associations are presented in Table S3. While *H4C13* (*β* = −0.03, *p_FDR_* = 6E‐02) is the top gene associated with brain amyloid burden, none of the individual genes met the pre‐determined FDR correction threshold[Table alz70982-tbl-0002] of 0.05. Five of the top gene associations in the grey60 module, shown in Table 3, are from the major histone gene cluster on chromosome 6, which is critical for gene regulation, and include the *H1‐5* (*HIST1H1B*) hub gene. Following gene‐set enrichment analysis, the grey60 module was enriched for genes involved in nucleosome assembly, nuclear division, and DNA damage pathways (Figure 2 and Table S4).

**TABLE 3:** Top individual gene associations with brain amyloid in grey60 module.

Ensembl ID	Gene	Chr	*β*	*p*	*p_FDR_*
ENSG00000287080	*H3C3*	6	−0.025	2.13E‐04	0.004
ENSG00000276410	*H2BC3*	6	−0.020	2.53E‐04	0.004
ENSG00000184357	*H1‐5*	6	−0.027	4.31E‐04	0.005
ENSG00000276368	*H2AC14*	6	−0.020	6.54E‐04	0.007
ENSG00000171848	*RRM2*	2	−0.023	1.06E‐03	0.007

*Note*: *P_FDR_* column contains *p* values corrected for 32 tests using the false discovery rate (FDR).

Abbreviation: chr, chromosome.

In sensitivity analyses covarying for p‐tau217 (*n* = 724), a strong blood biomarker of brain amyloidosis,^4^ the grey60 module association with amyloid burden was attenuated (*β* = 0.13, *p* = 0.944; Tables S5 and S6), suggesting that the gene co‐expression network cannot explain variance in brain amyloid burden above and beyond p‐tau217. That said, the low correlation between p‐tau217 and the grey60 module (Pearson's *R* = −0.12) suggests that this module does not represent the same biology as p‐tau217.

### 3.2 Cell‐type associations with brain amyloid burden

Results for the cell‐type analyses are presented in Tables S7 and S8. A higher fraction of activated NK (*β* = −0.45, *p* = 0.018) and activated CD4+ T cells (*β* = −0.17, *p* = 0.03) were related to lower amyloid deposition; however, these associations did not survive correction for multiple comparisons. There were no other cell‐type associations with brain amyloid.

## 4 DISCUSSION

We provided the largest whole‐blood transcriptomic analysis of brain amyloid burden in preclinical AD. Our results highlight a novel association between a gene module that includes the major histone gene cluster and the proportion of activated NK and CD4+ T cells in relation to brain amyloid burden. Together, these results highlight notable changes in the blood compartment in the preclinical stages of disease, suggesting gene regulation changes in the periphery are detectable long before the clinical manifestation of AD.

### 4.1 Histone genes show protective effect against amyloid deposition

The most striking finding from our network analysis was the strong inverse association between the histone module's expression and the brain amyloid burden. This module, primarily composed of histone genes from the major HIST1 cluster on chromosome 6, suggests higher peripheral expression of these genes may represent a blood‐based transcriptomic signature of brain amyloid deposition. One potential interpretation of our findings is that the HIST1 gene cluster association with amyloid burden reflects a response downstream of amyloid. Indeed, there is evidence that Aβ can induce histone changes in the brain,^23^ and numerous alterations to genes within this cluster are observed in the AD brain.^24^ It is possible that such alterations would be observable in the periphery. A second interpretation is that these *HIST1* alterations may be along a causal pathway. For example, multiple histone deacetylase inhibitors have been proposed as potential therapeutic targets for AD.^25^ While our work adds to the growing literature on histone gene alterations in AD, future mechanistic work will be required to clarify whether these alterations can be leveraged for emerging therapeutic or diagnostic approaches in AD.

### 4.2 Higher expression of a network of histone and DNA damage pathway genes

Our study identified a novel link between peripheral blood gene expression and brain amyloid burden in cognitively unimpaired individuals. This association is centered on a distinct gene‐network expression module primarily composed of histone genes (30 of 32) on chromosome 6. Four histone genes, *H1‐5*, *H3C3*, *H2BC3*, *H2AC14*, and one ribonucleotide gene *RRM2* (ribonucleotide reductase regulatory subunit M2), emerged as key genes driving this association, where *H1‐5* emerged as the hub gene for this module.

*H1‐5* encodes the linker H1 histone protein (also known as *H1B*, *H1F5*, *H1S‐3*, and *HIST1H1B*) that binds to the DNA between nucleosomes and helps condense DNA to form chromatin fiber. Beyond this canonical function to compact DNA, H1 protein is found in the cytoplasm of neurons and astrocytes, where it preferentially binds and aggregates filamentous amyloid‐like proteins Aβ_1‐42_ and α‐synuclein.^26^, ^27^, ^28^ Further, extracellularly released H1 from injured or dying neurons is a powerful chemoattractant and proinflammatory driver for microglia and astrocytes.^29^, ^30^, ^31^

*RRM2* is involved in the synthesis of deoxyribonucleotides, the building blocks of DNA, essential for DNA replication and damage repair.^30^, ^31^ In animal studies, reduced *RRM2* expression was associated with increased DNA damage and mitochondrial dysfunction,^32^ and both DNA damage and mitochondrial dysfunction have been implicated in the early pathogenesis of AD.

Chromosome 6 contains the largest cluster of histone genes known as *HIST1* (6p21‐6p22), which contains 55 genes,^33^, ^34^ including *H1‐5*, *H3C3*, *H2BC3*, and *H2AC1*. Epigenetic modifications, including histone modifications, for example, acetylation and methylation, can alter chromatin structure and accessibility, which in turn play a vital role in regulating gene expression. The dynamic organization of chromatin structure and epigenetic aberration is a key mechanism in epigenetic regulation in gene expression and is implicated in AD.^35^, ^36^ Epigenetic changes have been observed at early stages of AD, potentially leading to altered expression of genes involved in neuronal function, amyloid processing, and inflammation.^36^, ^37^, ^38^, ^39^, ^40^, ^41^, ^42^, ^43^

Additionally, proper chromatin structure is essential for efficient DNA repair. Age is the strongest risk factor for AD, and numerous studies have demonstrated that DNA damage accelerates with aging and AD. Alterations in the *HIST1* gene may affect chromatin accessibility, potentially hindering DNA repair mechanisms and making neurons vulnerable to damage as implicated in AD. These findings prioritize the *HIST1* genes; however, there is limited direct evidence linking *HIST1* gene variations on chromosome 6 to increased AD risk,^44^ so further research establishing causal link is needed.

Although we did not formally evaluate *APOE* interactions in the present manuscript, it is quite likely that peripheral transcriptomic signatures of brain amyloid burden may differ by genetic background. Future work should focus on a detailed evaluation of the modifying role of *APOE ε2* and *APOE* on brain amyloid burden.

The association between a histone gene module and brain amyloid underscores the potential of peripheral blood biomarkers to reflect brain pathology in AD and highlights the importance of epigenetic regulation in disease pathogenesis. Multi‐omics approaches in blood that integrate transcript, epigenetic, and proteomic alterations may be critical for subtyping the disease even in its earliest stages. Future studies investigating cell‐specific contributions to the observed blood gene expression changes and studies focused on uncovering the precise mechanisms connecting peripheral histone expression to brain amyloid burden are needed.

### 4.3 Activated NK and CD4+ T‐cell abundance relates to brain amyloid burden

Additionally, our study uncovered an association between activated NK and CD4+ T‐cell proportions in the blood with brain amyloid burden. Mounting evidence links inflammatory and immune alterations to AD, suggesting the role of the peripheral immune system in the asymptomatic AD period. NK cells are a type of cytotoxic lymphocyte participating in surveillance and response against intracellular pathogens and malignancy. Page et al.^45^ found activated peripheral NK cells in amnestic mild cognitive impairment but not in mild AD patients, suggesting an active immune response against an unknown aggressor. In AD mouse model studies, depletion of NK cells is also found to improve cognitive function,^46^ suggesting a detrimental role of NK cells with AD progression.

Traditionally, T cells, part of the adaptive immune system, were thought to be primarily located in the periphery (blood and lymphoid organs). However, recent research has identified an important role T cells play in neuroprotection and neuroinflammation.^47^ Activated CD4+ T cells or helper T cells are best known for assisting other immune cells in mounting effective immune responses and, once activated, can differentiate into various subtypes (e.g., Th1, Th2, Th17, and Treg) that secrete different cytokines and perform distinct functions. Studies have shown CD4+ T cells enhance NK cell function^48^ in response to viral infection. That said, the relationship between blood and brain innate and adaptive immune cell abundance in the preclinical stages of AD remains unknown, so future studies that elucidate this complex interaction between compartments will be critical for the proper interpretation of peripheral changes in immune cell abundance in preclinical AD.

### 4.4 Strengths and weaknesses

This study has several strengths, including the large sample size, comprehensive characterization of the cohort including PET imaging for brain amyloid burden, and robust statistical approach with appropriate corrections for multiple comparisons. Despite these strengths, there were some limitations. The cohort included in the A4 Study is highly educated, predominantly non‐Hispanic White, and does not include participants with overt dementia. While methods such as CIBERSORTx aid in deconvolving cellular contributions, our analysis was limited to whole‐blood data, limiting our ability to make cell‐type‐specific inferences within this analysis. Moreover, while we are focused on correlations between blood expression and brain amyloid burden, many processes outside of the brain contribute to blood expression changes, complicating the interpretation and directionality of the reported correlations in this study. Despite these limitations, the study does demonstrate detectable blood transcript alterations that are related to brain amyloid burden even at the earliest stages of disease.

## 5 CONCLUSIONS

This large study of blood gene expression and brain amyloid burden implicates DNA damage and activated memory T cells as novel pathways in preclinical AD. Future work will evaluate the ability of such gene network alterations to improve preclinical prediction of future cognitive decline.

## CONFLICT OF INTEREST STATEMENT

Dr. Hohman is on the scientific advisory board for Circular Genomics, serves as deputy editor for Alzheimer's & Dementia: TRCI, senior associate editor for Alzheimer's & Dementia. Dr. Buckley is chair of the Sex & Gender ISTAART Professional Interest Area and on the Women's Health steering committee. Dr. Koran is on the SNMMI Brain Imaging Council, Brain Imaging Outreach Group. Dr. Aisen consults for Merck, Roche, Genentech, AbbVie, Immunobrain, Biogen, Arrowhead, and Checkpoint and has research collaboration with Eisai and CogRx. Dr. Sperling consults for AbbVie, AC Immune, Acumen, Alector, Apellis, Biohaven, Bristol Myers Squibb, Genentech, Janssen, NervGen, Oligomerix, Prothena, Roche, Vigil Neuroscience, Ionis, and Vaaxinity. Other authors have nothing to disclose. Author disclosures are available in the Supporting Information.

## CONSENT STATEMENT

Informed consent was obtained from all participants as part of the parent study, and the Vanderbilt University Institutional Review Board approved the secondary analysis of these data for the present manuscript.

## Supporting information



Supporting Information

Supporting Information

## Data Availability

All the data used in these analyses are available for download from the following data repositories: the Laboratory for Neuroimaging's Image and Data Archive (LONI IDA) (ida.loni.usc.edu), Alzheimer's Clinical Trials Consortium (ACTC, https://www.a4studydata.org/), the Global Alzheimer's Association Interactive Network platform (GAAIN) (www.gaain.org), and Synapse (https://www.synapse.org/Synapse:syn61250768).
